# Ubiquitin-specific protease-44 inhibits the proliferation and migration of cells via inhibition of JNK pathway in clear cell renal cell carcinoma

**DOI:** 10.1186/s12885-020-6713-y

**Published:** 2020-03-12

**Authors:** Jiangqiao Zhou, Tianyu Wang, Tao Qiu, Zhongbao Chen, Xiaoxiong Ma, Long Zhang, Jilin Zou

**Affiliations:** Department of Organ Transplantation, Renmin Hospital of Wuhan University, Wuhan University, 99 ZiYang Road, Wuhan, 430060 China

**Keywords:** USP44, Clear cell renal cell carcinoma, Proliferation, JNK

## Abstract

**Background:**

Clear cell renal cell carcinoma (ccRCC) is the most common form of adult kidney cancer. Ubiquitin-specific protease (USP)44 has been reported to be involved in various cancers. We investigated the function, role and molecular mechanism of USP44 in ccRCC.

**Methods:**

Data obtained from the Cancer Genome Atlas Data Portal and Gene Expression Omnibus database were analyzed to uncover the clinical relevance of USP44 expression and tumor development. USP44 function in the proliferation and migration of tumor cells was assessed by cellular and molecular analyses using ccRCC lines (786-O cells and Caki-1 cells).

**Results:**

USP44 showed low expression in ccRCC cancer tissues compared with that in normal tissue. USP44 expression was negatively correlated with tumor stage, tumor grade, and patient survival. USP44 overexpression inhibited the proliferation and migration of 786-O cells and Caki-1 cells significantly. USP44 overexpression also prohibited cell proliferation by upregulating expression of P21, downregulating cyclin-D1 expression, and inhibiting cell migration by downregulating expression of matrix metalloproteinase (MMP)2 and MMP9. USP44 knockdown enhanced the proliferation and migration of 786-O cells and Caki-1 cells. USP44 function in inhibiting the proliferation and migration of 786-O cells and Caki-1 cells was associated with phosphorylation of Jun N-terminal kinase (JNK).

**Conclusion:**

USP44 may be a marker in predicting ccRCC progression. Inhibition by USP44 of the proliferation and migration of 786-O cells and Caki-1 cells is dependent upon the JNK pathway.

## Background

Renal cell carcinoma (RCC) is represents 80–90% of adult kidney cancers. RCC incidence varies geographically, with the highest incidence being documented in developed countries [1]. Based on recent guidelines, the most efficacious treatment for early-stage clear cell renal cell carcinoma (ccRCC) is surgery and targeted therapy [2]. Unfortunately, the major cause of death for most ccRCC patients is the metastasis and recurrence of tumor cells [3]. Several new biomarkers have been explored to diagnose and predict the occurrence and development of ccRCC [4–6].

Chromosomal instability, leading to aneuploidy, is one of the hallmarks of human cancers [7]. Ubiquitin-specific protease (USP)44 is located at 12q22 and encodes a 712-kD amino acid. USP44 is a member of a family of deubiquitinating enzymes and has an important role in human cancers [8]. USP44 regulates the separation and positioning of centrosomes, and the geometry of mitotic spindles [9]. USP44 can stabilize the protein expression of protectin in the cycle of healthy cells until all the chromosomes match correctly with spindle fibers and prevent immature mitosis. By inhibiting USP44 expression in mice, the proportion of aneuploid cells and chromosomal instability can be increased significantly, making them more prone to malignant transformation [10, 11]. However, Zou and colleagues showed that USP44 overexpression promotes the malignancy of glioma [12].

However, the function and mechanism of action of USP44 in ccRCC have not been clarified, a knowledge gap we aimed to fill in the present study.

## Methods

### Reagents

Antibodies against Flag (catalog number: M185-3 L) and β-actin (M177–3) were purchased from Medical Biological Laboratories (Nagoya, Japan). Antibodies against matrix metalloproteinase (MMP)9 (A0289) were obtained from ABclonal (Woburn, MA, USA). Antibodies against P21 (2947), cyclin D1 (2978), c-Jun N-terminal kinase (JNK; 9252), phosphorylated (p)-JNK (4668), protein kinase B (AKT; 4691), p-AKT (4060), p38 (9212), p-p38 (4511), extracellular signal-regulated kinase (ERK; 4695) and p-ERK (4370) were purchased from Cell Signaling Technology (Danvers, MA, USA).

The JNK inhibitor JNK-IN-8 (HY-13319, MCE, USA) was dissolved in dimethyl sulfoxide (DMSO) and diluted into a 0.5-μM working solution with complete culture medium, and the same amount of DMSO was set as the control.

### Bioinformatics analysis

Bioinformatics analysis was undertaken in accordance with the work of Jiangqiao and collaborators [13]. ccRCC’s gene sequence tertiary count data samples and clinical information are obtained through TCGA data portal. DESeq2 within the R Project for Statistical Computing (Vienna, Austria) was used to standardize counting data and analyze differentially expressed genes between cancer samples and normal samples. Standardized data were used primarily to analyze the visual expression, stage, grade and survival correlation of USP44 in ccRCC and adjacent non-cancerous tissues. According to USP44 expression, clinical samples of ccRCC were divided into two groups for analyses.

Kaplan–Meier survival curves were used to show the differences in overall survival between patients with high expression of USP44 and cases with low expression of USP44. Simultaneously, we calculated the correlation between USP44 expression and the age, sex, tumor stage and tumor grade of the patient through T-text, and the obtained data were visualized through ggplot2 within the R Project for Statistical Computing.

### Cells

The human ccRCC line 786-O (CRL-1932) was purchased from BeNa Culture Collection (Manassas, VA, USA). Cells were cultured in Dulbecco’s modified Eagle’s medium (DMEM; C11995500BT; Billings, MT, USA) [14]. Caki-1 (a cell line of human ccRCC that metastasizes to the skin) was purchased from the Chinese Academy of Sciences Cell Bank (TCHu135; Beijing, China) and was cultured in McCoy’s 5A culture medium (L630; Basal Media, Saint Louis, MO, USA) [15]. Then, 10% fetal bovine serum (FBS; F05–001-B160216; One Biotechnology, Sarasota, FL, USA), penicillin (100 U/mL) and streptomycin (100 μg/mL) were added to DMEM and McCoy’s 5A. 786-O cells and Caki-1 cells were cultured in a humidified environment at 37 °C containing 5% carbon dioxide.

### Lentivirus of overexpressed USP44, and construction and production of short hairpin (sh)RNA USP44 lentivirus

An overexpressed vector with a flag tag and shRNA vectors of the USP44 (homo) gene were designed and constructed according the method described by Jiangqiao and colleagues [13]. The gene registration number is NM_001347937.1. pHAGE-3xflag was used as the carrier. The primers were h-USP44-NF, AAACGATTCAGGTGGTCAGG, h-USP44-NR, and AGTGTACCCAGAACCCTCCT. The sequence of pLKO.1-h-USP44-shRNA1 was CGGATGATGAACTTGTGCAAT. The sequence of pLKO.1-h-USP44-shRNA2 was GCACAGGAGAAGGATACTAAT.

### Cell counting kit (CCK)8 assay

Cell viability was examined using a CCK-8 kit following manufacturer (44,786; Dojindo, Tokyo, Japan) protocols [13]. 786-O cells and Caki-1 cells were inoculated in 96-well plates (167,008; Thermo Scientific, Waltham, MA, USA). After cells had adhered to the plate, they were cultured further for 0, 12, 24, 36, 48, and 60 h, respectively. CCK8 reagents (10 μL) were added and absorbance at 450 nm measured.

### 5-bromo-2′-deoxyuridine (BrdU) experiment

The BrdU experiment was undertaken according to manufacturer (11,647,229,001; Roche, Basel, Switzerland) instructions [13]. 786-O cells and Caki-1 cells were inoculated in 96-well plates. After 24 h and 48 h, the BrdU experiment was carried out.

### Wound-healing test

786-O cells and Caki-1 cells were inoculated into six-well plates (140,675; Thermo Scientific) at 3 × 10^5^ cells per well and incubated overnight. After that, the original culture medium was replaced with DMEM containing mitomycin (10 μg/mL). Then, cells were cultured for 12 h. Cells were wounded with a pipette tip and photographs taken immediately (0 h) as well as 6 h and 12 h after wounding. Then, the Cell Migration Index was calculated using the following formula:

Cell Migration Index = (wound width at 0 h – wound width at 6 h or 12 h) × 100/wound width at 0 h.

### Cell-migration assay

Healthy 786-O cells and Caki-1 cells were resuspended in DMEM or McCoy’s 5A. Then, they were plated at 3 × 10^4^ cells/well (786-O) or 5 × 10^4^ cells/well (Caki-1) in the upper compartment of a Transwell™ chamber (3421; Corning, Corning, NY, USA). Meanwhile, DMEM containing 600 μL of 2% FBS or 600 μL of 10% FBS was added to the lower chamber, respectively. Cells were cultured for 2 h or 3 h (786-O) or 10 h or 24 h (Caki-1) with phosphate-buffered saline. Then, 600 μL of 4% paraformaldehyde solution was used to fix cells for 15 min at room temperature, and 600 μL of 0.1% crystal violet (548–62-9; Xinkang,Hubei) was used to stain cells for 2 h at 37 °C. Images were acquired under a microscope. The number of positively stained cells reflected the cell-migration ability.

### Western blotting

Proteins were extracted from 786-O cells and Caki-1 cells according to standard protocols. Meanwhile, protease inhibitors (04693132001; Roche) and phosphatase inhibitors (4,906,837,001; Roche) were added. Protein concentrations were determined using a Bicinchoninic Acid Protein Assay kit (23,225; Thermo Fisher Scientific). Briefly, we separated protein samples by sodium dodecyl sulfate-polyacrylamide gel electrophoresis on 12.5% gels, and then transferred them to nitrocellulose membranes. We blocked the nitrocellulose membranes using 5% nonfat dry milk in TBS-T buffer and incubated them overnight with primary antibody at 4 °C. After rinsing the blots extensively with TBS-T buffer, incubation with secondary antibodies for 1 h was undertaken. We applied a ChemiDoc™ XRS+ gel-imaging system (Bio-Rad Laboratories, Hercules, CA, USA) to detect the target bands.

### Reverse transcription-polymerase chain reaction (RT-PCR)

The total mRNA of 786-O and Caki-1 cell lines was extracted with TRIzol® Reagent (15596–026; Invitrogen, Carlsbad, CA, USA). Then, total RNA was reverse-transcribed into complementary (c)DNA using a Transcriptor First Strand cDNA Synthesis kit (04896866001; Roche) according to manufacturer instructions. SYBR® Green (04887352001; Roche) was used to quantify the PCR-amplification products. mRNA expression of target genes was normalized to that of β-actin expression. All the primer information is in Table 1.

**Table 1:** Primers for qPCR detection

Gene name	Forward primer (Human)	Reverse primer (Human)
USP44	AAACGATTCAGGTGGTCAGG	AGTGTACCCAGAACCCTCCT
P21	TGGAGACTCTCAGGGTCGAAA	TTCCTCTTGGAGAAGATCAGCC
CyclinD1	CAGATCATCCGCAAACACGC	AGGCGGTAGTAGGACAGGAA
MMP2	CCGTCGCCCATCATCAAGTT	CCGCATGGTCTCGATGGTAT
MMP9	TTTGAGTCCGGTGGACGATG	TTGTCGGCGATAAGGAAGGG
β-ACTIN	CATGTACGTTGCTATCCAGGC	CTCCTTAATGTCACGCACGAT

### Statistical analyses

Data are the mean ± standard error. We used SPSS v19.0 (IBM, Armonk, NY, USA) for statistical analyses. The Student’s *t*-test was used to analyze all data. *P* < 0.05 was considered significant.

## Results

### USP44 expression is deceased in ccRCC tissue and is correlated with the tumor stage, tumor grade. And patient survival

**Fig. 1:**
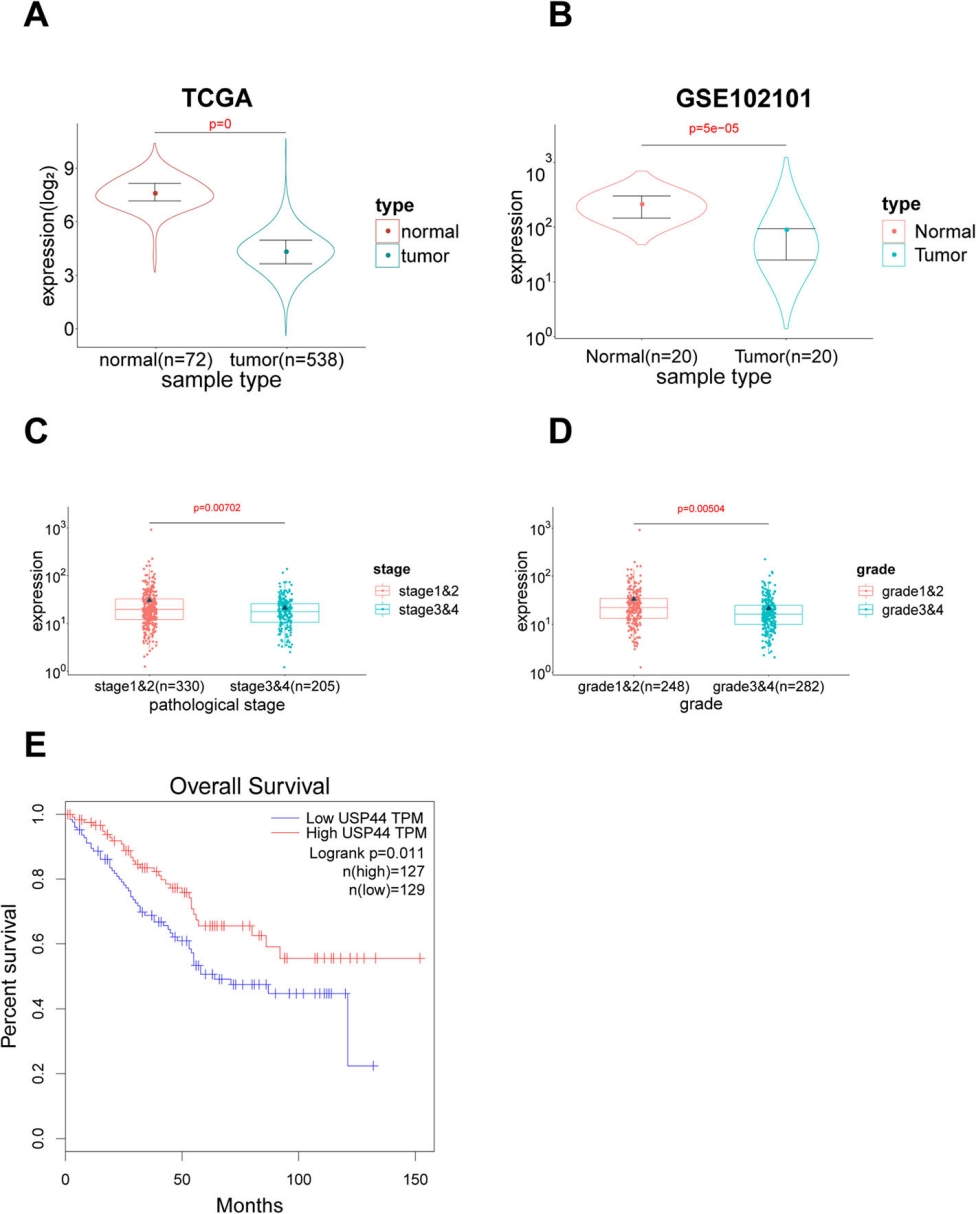
USP44 is involved in the occurrence and development of ccRCC. **a** Expression of USP44 mRNA of normal kidney tissue and ccRCC according to the results from the TCGA Data Portal. **b** Expression of USP44 mRNA of normal kidney tissue and ccRCC according to the results from the Gene Expression Omnibus (GEO) database. **c** Expression of USP44 mRNA in ccRCC cancer tissues at different tumor stages. **d** Expression of USP44 mRNA in ccRCC cancer tissues at different tumor grades. **e** Kaplan–Meier curve for ccRCC patients with low and high expression of USP44

Analyses of information from the TCGA Data Portal demonstrated that USP44 expression was significantly lower in ccRCC specimens than that in normal tissues (Fig. 1a). Data analyses from the Gene Expression Omnibus (GEO) 102,101 database confirmed this result (Fig. 1b). Relationship between the expression of USP44 and Clinicopathological characteristics in Table 2. Subsequently, a subgroup analysis was undertaken based on the stage and grade of ccRCC. USP44 expression was closely related to the stage and grade of ccRCC (Fig. 1c, d). With an increment in stage and grade, USP44 expression showed a gradual decrease. USP44 expression was closely related to patient survival (Fig. 1e). Based on these results, USP44 might be a potential marker to predict ccRCC progression, and play an important part in ccRCC progression.

**Table 2:** Relationship between the expression of USP44 and Clinicopathological characteristics

Category	Subcategory	Cases	USP44 expression mean rank	*P* value
Age	≤60	249	31.02941	0.05706
	> 60	288	23.13299	
Gender	Male	352	27.92778	0.57303
	Female	186	24.54313	
Grade	I-II	248	33.035699	0.00504
	III-IV	282	21.315288	
Stage	T1-T2	348	29.613193	0.01157
	T3-T4	190	21.52742	
Nodes	N0	240	23.622062	0.0041
	N1	16	15.18371	
Metastasis	Yes	427	27.59757	0.00019
	No	78	19.761654	

### USP44 overexpression inhibits proliferation of 786-O cells and Caki-1 cells

**Fig. 2:**
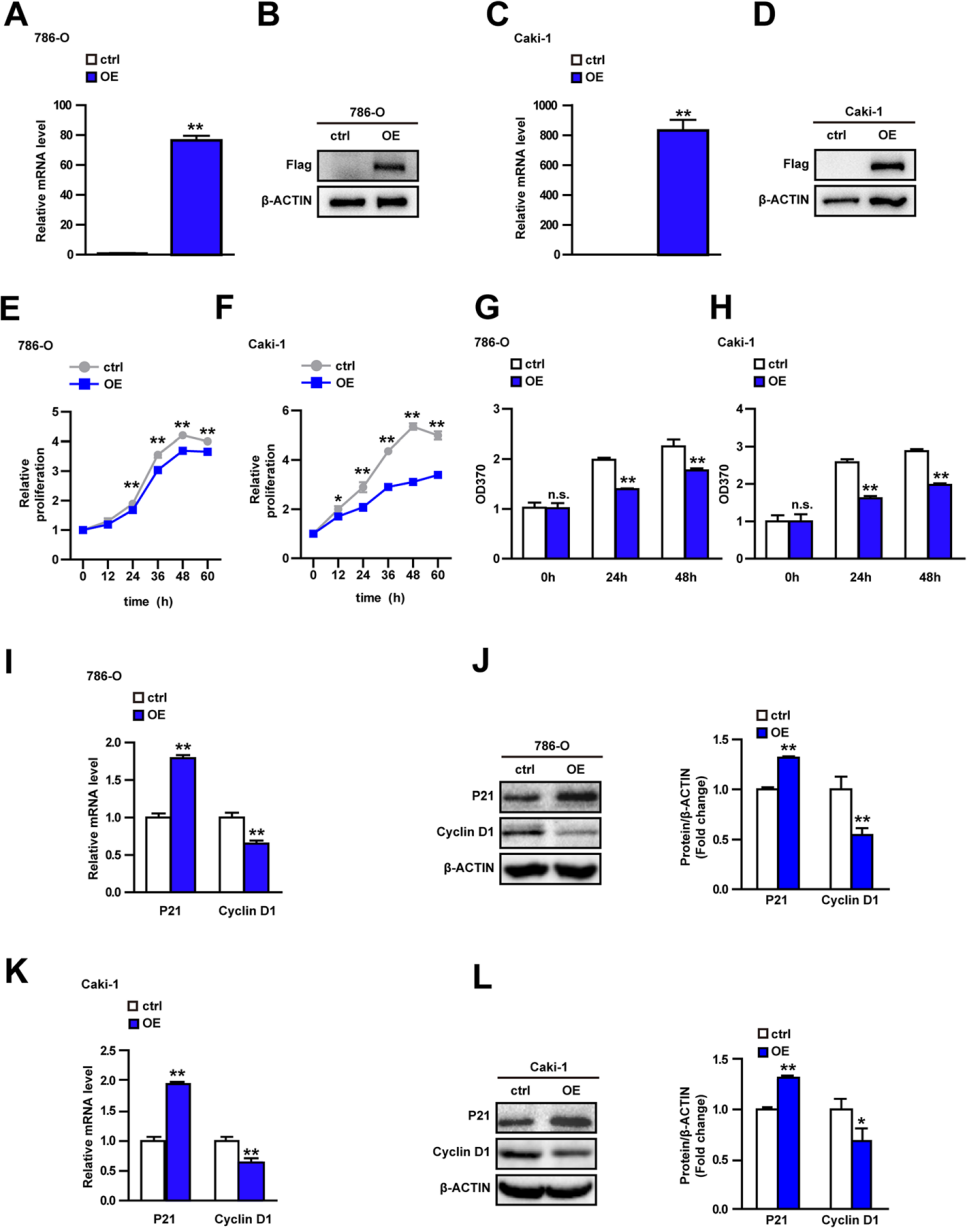
USP44 overexpression inhibits proliferation of 786-O cells and Caki-1 cells. **a**, **c** mRNA expression of USP44 in control (ctrl) and overexpression (OE) groups of 786-O cells (**a**) and Caki-1 cells (**c**). **b**, **d** Protein expression of FLAG in ctrl and OE groups of 786-O cells (**b**) and Caki-1 cells (**d**). The recombinant FLAG-USP44 fusion protein was constructed, so detection of FLAG expression reflected USP44 expression. (cropping of blots). **e**, **f** Relative proliferation of ctrl and OE groups of 786-O cells (**e**) and Caki-1 cells (**f**) in the CCK8 assay. **g**, **h** Absorbance at 370 nm in ctrl and OE groups of 786-O cells (**g**) and Caki-1 cells (**h**) in the BrdU experiment. **i**, **k** mRNA expression of P21 and cyclin D1 in ctrl and OE groups of 786-O cells (**i**) and Caki-1 cells (**k**). **j**, **l** Protein expression of P21 and cyclin D1 in ctrl and OE groups of 786-O cells (**j**) and Caki-1 cells (**l**). (cropping of blots). *P < 0.05, **P < 0.01 vs. the ctrl group. Data are the mean ± SD

We wished to explore the effect of USP44 in vitro. 786-O cells and Caki-1 cells show different metastatic and invasive abilities in the ccRCC model, so we chose these two cell lines for experiments. Overexpressed stable cell lines were obtained by viral infection of USP44 in 786-O cells and Caki-1 cells (Fig. 2a–d). The viability and proliferation potential of cells was evaluated through the CCK8 assay and BrdU experiment. In comparison with negative controls, USP44 overexpression inhibited the viability of these two lines significantly (Fig. 2e, f). To explore further the direct influence of USP44 on ccRCC proliferation, we labeled proliferating cells with BrdU in cells showing overexpression of USP44 and control cells. USP44 overexpression reduced the BrdU-absorption capacity of 786-O cells and Caki-1 cells significantly (Fig. 2g, h), which demonstrated that USP44 can inhibit ccRCC proliferation. Studies have shown that expression of cyclin D1 and P21 is closely related to tumor occurrence, and that they are markers of proliferation of tumor cells [16, 17]. The main function of cyclin D1 is to promote cell proliferation by regulating the cell cycle, which is closely related to the occurrence of tumors and is a marker of proliferation of tumor cells (including ccRCC) [18]. P21 expression is closely related to inhibition of tumor cells and can coordinate the relationship between the cell cycle, DNA replication and DNA repair by inhibiting the activity of cyclin-dependent kinase complexes [19]. USP44 expression was positively correlated with expression of the gene and protein of P21, and negatively correlated with expression of the gene and protein of cyclin D1 (Fig. 2i–l). Taken together, these results demonstrated that USP44 inhibited proliferation of 786-O cells and Caki-1 cells.

### UPS44 overexpression inhibits migration of 786-O cells and Caki-1 cells

**Fig. 3:**
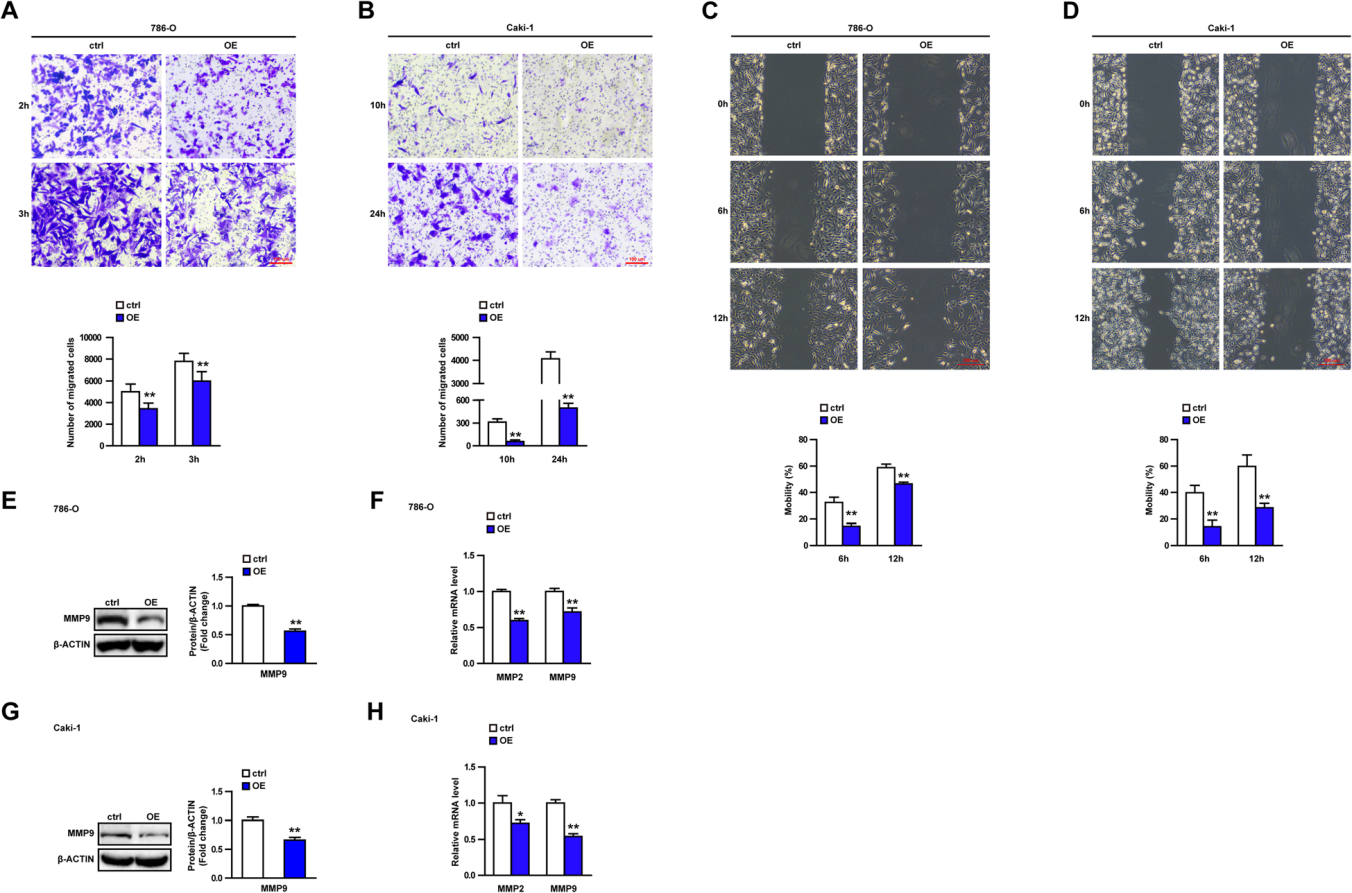
USP44 overexpression inhibits migration of 786-O cells and Caki-1 cells. **a**, **b** Image of the Transwell™ result of 786-O cells (**a**) and Caki-1 cells (**b**) and the histogram shows the statistical analysis of migrated cells. **c**, **d** Wound-healing test for 786-O cells (**c**) and Caki-1 cells (**d**). The histogram shows the statistical analysis of cell mobility. **e**, **g** Protein expression of MMP9 in control (ctrl) and overexpression (OE) groups of 786-O cells (**e**) and Caki-1 cells (**g**). (cropping of blots). **f**, **h** mRNA expression of MMP2, MMP9 in ctrl and OE groups of 786-O cells (**f**) and Caki-1 cells (**h**). *P < 0.05, **P < 0.01 vs. the ctrl group. Data are the mean ± SD

We conducted a series of experiments to investigate if USP44 overexpression inhibited the migration of 786-O cells and Caki-1 cells. First, we used Transwells to evaluate the effect of USP44 overexpression on cell migration. We found that USP44 overexpression slowed down the migration of 786-O cells and Caki-1 cells significantly (Fig. 3a, b), which was consistent with our expectation. Because the two types of tumor cells we used have different migration abilities, USP44 overexpression slowed down the migration ability of 786-O cells at the early stage (2 h, 3 h), and slowed down the migration ability of Caki-1 cells at the late stage (10 h, 24 h).

Next, we undertook wound-healing experiments to confirm the migration effect of USP44. To avoid the effect of cell proliferation on cell migration, mitomycin was administered before wound-healing experiments. USP44 overexpression slowed down the migration of 786-O cells and Caki-1 cells significantly (Fig. 3c, d). MMP2 and MMP9 are closely related to the blood-vessel formation, growth and metastasis of tumors [20]. MMP2 and MMP9 have been recognized as markers of the migration and metastasis of ccRCC lines [21]. USP44 overexpression down-regulated expression of the mRNA and protein of MMP2 and MMP9 in 786-O cells and Caki-1 cells (Fig. 3e–h). Collectively, these results demonstrated that USP44 inhibited the migration of 786-O cells and Caki-1 cells.

### UPS44 knockdown promotes the proliferation and migration of Caki-1 cells

**Fig. 4:**
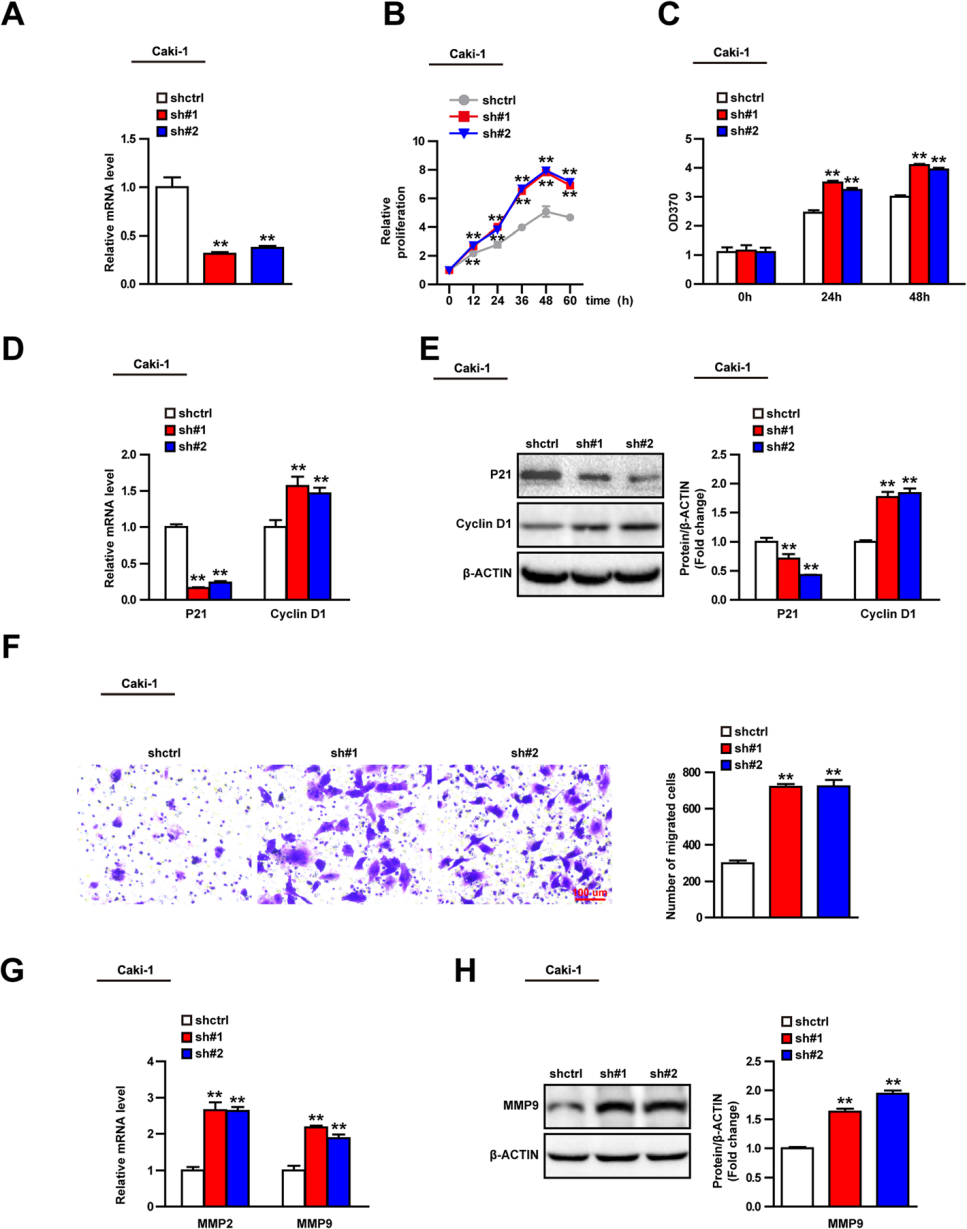
USP44 knockdown promotes the proliferation and migration of Caki-1 cells. **a** mRNA expression of USP44 in short hairpin control (shctrl) and shUSP44 groups of Caki-1 cells. **b** Relative proliferation of shctrl and shUSP44 groups of Caki-1 cells in the CCK8 assay. **c** Absorbance at 370 nm in shctrl and shUSP44 groups of Caki-1 cells in the BrdU experiment. **d**, **e** Expression of mRNA and protein of P21 and cyclin D1 in shctrl and shUSP44 groups of Caki-1 cells. (cropping of blots). **f** Image of the Transwell™ result in shctrl and shUSP44 groups of Caki-1 cells, and the histogram shows the number of migrated cells. **g**, **h** Expression of mRNA and protein of MMP9 and MMP2 mRNA expression in shctrl and shUSP44 groups of Caki-1 cells. (cropping of blots). **P < 0.01 vs. the ctrl group. Data are the mean ± SD

We attempted to verify the role of USP44 in tumor cells by silencing USP44 expression with shRNAs. Two shRNAs were constructed to silence USP44 expression in Caki-1 cells (Fig. 4a). Consistent with our expectation, USP44 knockdown promoted cell proliferation significantly according to the CCK-8 assay and BrdU experiments (Fig. 4b, c). USP44 knockdown inhibited P21 expression and upregulated expression of cyclin D1 (Fig. 4d, e). The cell-migration assay showed that USP44 deficiency promoted the migration of Caki-1 cells (Fig. 4f), which was associated with upregulation of expression of MMP2 and MMP9 (Fig. 4g, h). These results confirmed that USP44 knockdown enhanced the proliferation and migration of Caki-1 cells.

### USP44 suppressed the JNK signaling pathway in ccRCC

**Fig. 5:**
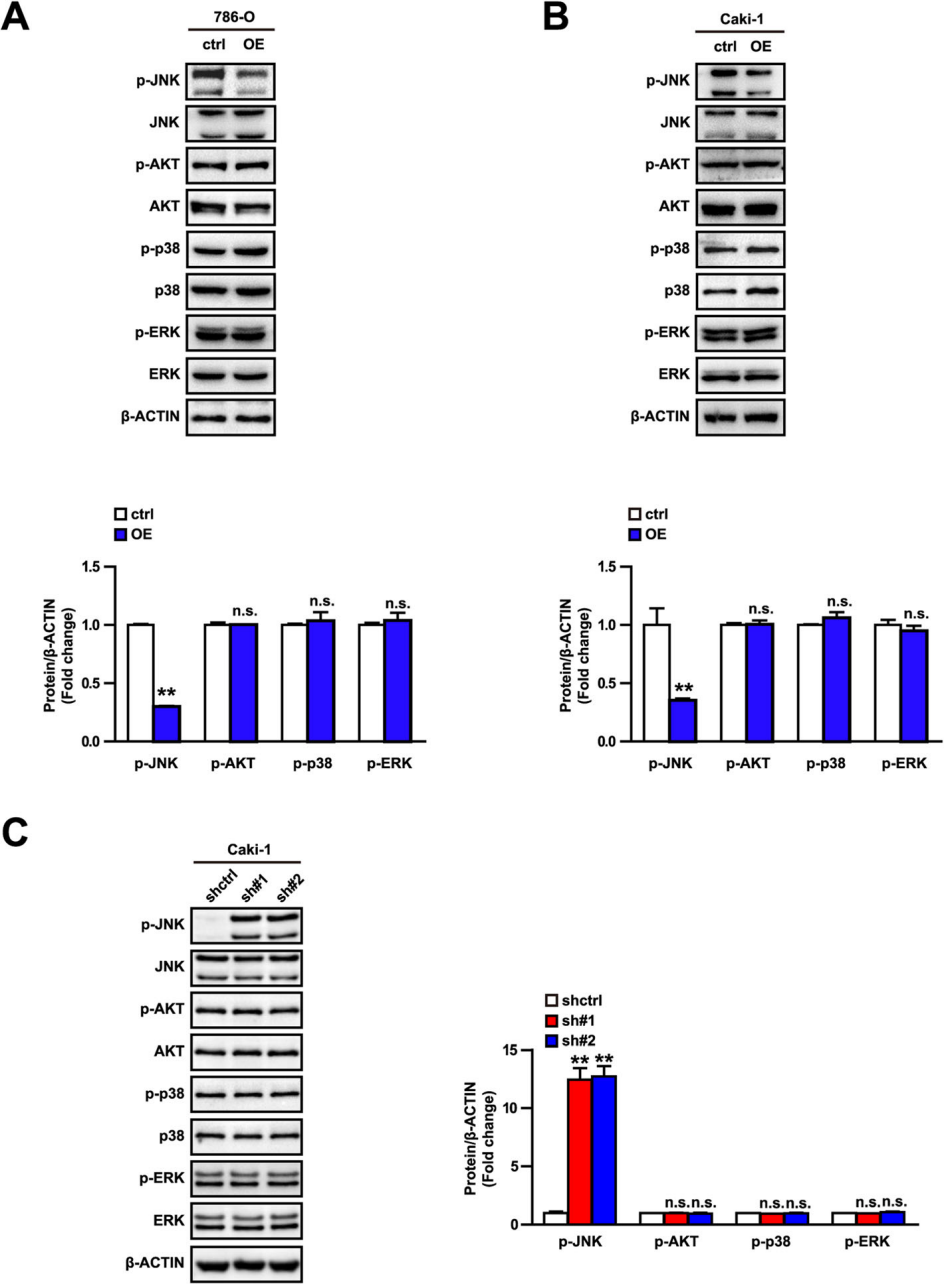
The JNK signaling pathway is related to the regulation of USP44 function in ccRCC development. **a**, **b** Western blots for molecules in the MAPK signaling pathway (JNK,AKT,p38,ERK) in control (ctrl) and overexpression (OE) groups of 786-O cells (**a**) and Caki-1 cells (**b**). (cropping of blots). **c** Western blots of molecules in the MAPK signaling pathway (JNK,AKT,p38,ERK) in short hairpin control (shctrl) and shUSP44 groups of Caki-1 cells. (cropping of blots). **p < 0.01 vs. the ctrl group; n.s. not significant vs. the shctrl group. Data shown are the mean ± SD

The AKT and mitogen activated protein kinase (MAPK) signaling pathways have important roles in the occurrence and development of malignant tumors [22]. To explore how USP44 regulates the proliferation and migration of tumor cells, we measured the activation of AKT, JNK, p38, and ERK signal pathways in USP44-overexpression and control groups. USP44 overexpression decreased the level of JNK, but not that of AKT, p38 or ERK, compared with control cells in both cell lines (Fig. 5a, b). JNK expression was promoted if USP44 expression was knocked down, but no effect was observed on expression of AKT, p38 or ERK (Fig. 5c). The results stated above suggest that the JNK signaling pathway participated in the USP44 function of regulating proliferation of 786-O cells and Caki-1 cells.

### The promotional effect of USP44 knockdown on the proliferation and migration of 786-O cells and Caki-1 cells was dependent upon the JNK pathway

**Fig. 6:**
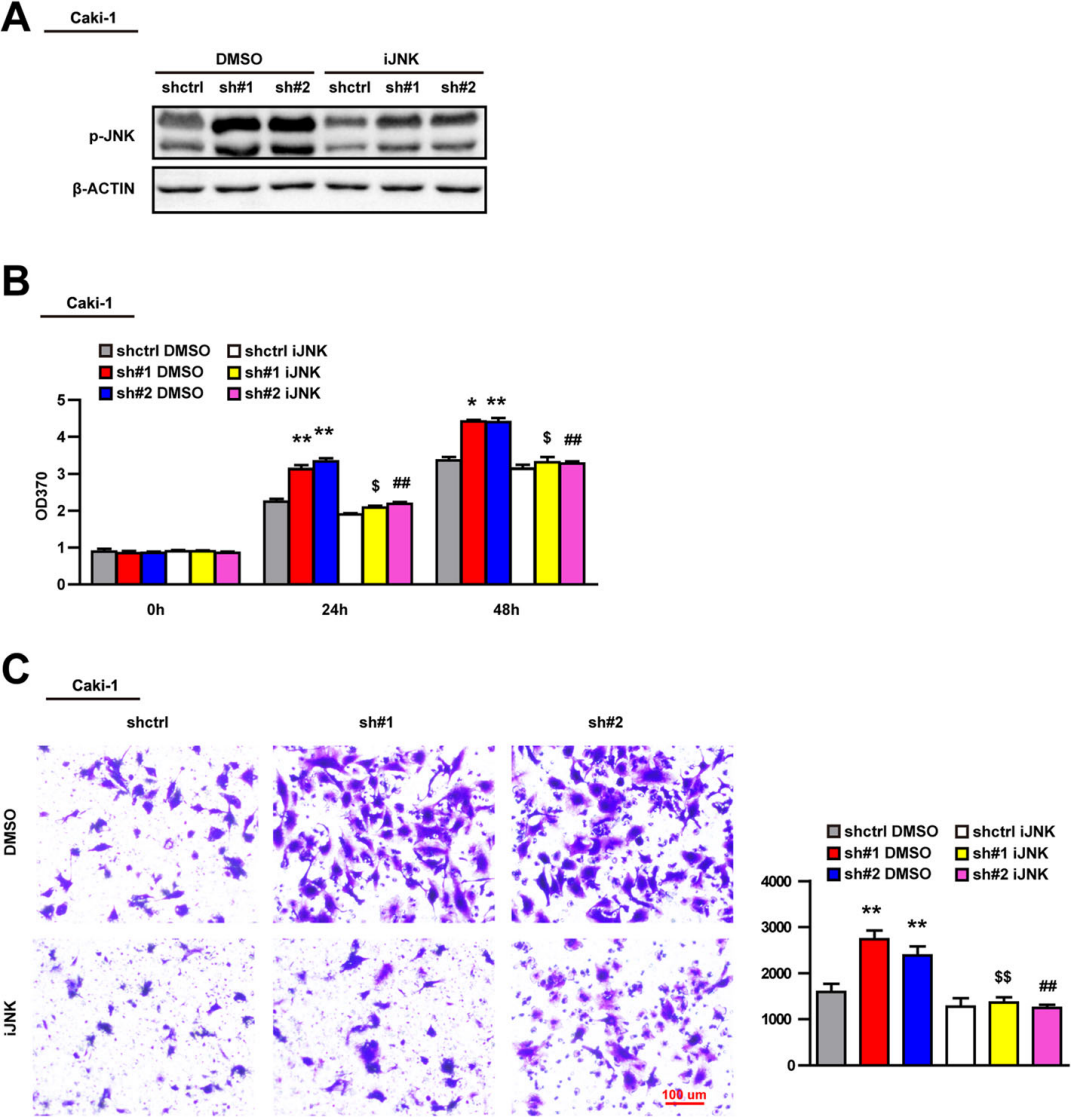
USP44 knockdown promotes the proliferation and migration of Caki-1 cells through JNK activity. **a** Western blotting showed the p-JNK level of Caki-1 cells in short hairpin control (shctrl) and shUSP44#1, and shUSP44#2 groups with or without a JNK inhibitor. (cropping of blots). **b** BrdU experiment showing the relative proliferation index of Caki-1 cells in shctrl and shUSP44#1, and shUSP44#2 groups with or without a JNK inhibitor. **c** Image of the Transwell™ result for Caki-1 cells in the shctrl and shUSP44#1, and shUSP44#2 groups with or without a JNK inhibitor, and the histogram shows the number of migrated cells. *p < 0.05 vs. the shctrl DMSO group; **p < 0.01 vs. the shctrl DMSO group; $*p* < 0.05 vs. the sh#1 DMSO group; $$*p* < 0.01 vs. the sh#1 DMSO group; ##*p* < 0.01 vs. the sh#2 DMSO group

To verify further whether the role of USP44 in ccRCC progression was dependent upon the JNK pathway, we blocked JNK activation via a JNK inhibitor and examined the proliferation and migration of 786-O cells and Caki-1 cells (Fig. 6a). Results showed that the ability of USP44 knockdown to promote the proliferation and migration of 786-O cells and Caki-1 cells was reduced significantly after treatment with a JNK inhibitor. Hence, USP44 regulated the proliferation and migration of 786-O cells and Caki-1 cells through the JNK signaling pathway (Fig. 6b, c).

## Discussion

Several studies have demonstrated that the molecular mechanism of ccRCC is closely related to apoptosis, autophagy, hypoxia metabolism and immune imbalance [23]. However, the mechanism of pathogenesis and metastasis of ccRCC have not been elucidated.

The spindle assembly checkpoint (SAC) is an important mechanism to ensure mitosis. An abnormality of the SAC is a key step in the development of aneuploidy and even tumors. Holland and colleagues reported that the important regulatory proteins of the SAC deubiquitinase USP44 were closely associated with tumors [24].

We explored the role of USP44 as a tumor marker based on information from the TCGA Data Portal and GEO 102010 database. Results showed that USP44 had low expression in tumor tissues and correlated with the pathologic stage and grade of tumors. Patients with high USP44 expression showed good survival benefits. These results suggest that USP44 may be a good biomarker to predict ccRCC progression.

Some studies have suggested that USP44 overexpression promotes tumor development, whereas other studies have indicated that USP44 inhibits proliferation of tumor cells [10, 11, 25, 26]. Thus, we examined the effect of USP44 on ccRCC proliferation. Using 786-O cells and Caki-1 cells, we showed that USP44 overexpression inhibited proliferation of these two cell lines. The genes associated with proliferation of these two cell lines were also regulated by USP44 overexpression.

The metastatic potential of ccRCC is the main factor leading to the death of affected patients [27]. Treatment of metastatic ccRCC has changed considerably over recent years [28]. The US Food and Drug Administration has approved agents to treat metastatic ccRCC, including immunotherapeutic drugs, antiangiogenic agents, and mammalian target of rapamycin (mTOR) inhibitors [1, 29]. Nevertheless, even with these treatments, many patients with metastatic ccRCC have very short survival. We demonstrated that USP44 overexpression inhibited migration of tumor cells through wound-healing and cell-migration experiments. To avoid the effect of cell proliferation on cell migration, mitomycin was administered before wound-healing experiments.

The MMP family are involved in breakdown of the extracellular matrix in health and disease (e.g., metastasis) [20]. MMP2 and MM9 are closely related to the invasion and metastasis of several types of tumor cells [30]. Our data showed that USP44 overexpression in 786-O cells and Caki-1 cells was a reminder that ccRCC metastasis was related to expression of MMP2 and MMP9. Based on the results from Caki-1 cells with USP44 silencing by shRNAs, we demonstrated that USP44 inhibits ccRCC progression in reverse.

Whether a deubiquitinating enzyme has a role in promoting or inhibiting cancer is closely related to the function of its substrate protein [31]. Substrate molecules regulate several tumor-associated signaling pathways: p53, nuclear factor-kappa B, Wnt, transforming growth factor-β, and histone epigenetic modifications. These signaling pathways interact with each other. Upregulation of USP expression in tumor cells often suggests that its substrate protein can promote the malignant progression of cancer cells [32]. Downregulated expression of a USP suggests that its substrate is usually a tumor suppressor. Each USP has multiple substrates, and the same substrates may be regulated by multiple USPs [33]. Therefore, the regulatory network of a USP on a tumor-cell signaling pathway is extremely complex.

PI3K/AKT is a serine/threonine protein kinase involved in tumorigenesis (including ccRCC) [34]. If cells are stimulated by extracellular signals, PI3K activates AKT, and the latter further activates its downstream factor mTOR. The MAPK signaling pathway has crucial roles in the occurrence, development, treatment and prognosis of malignant tumors [35]. The downstream signaling pathway includes JNK, ERK and p38, which are associated with the growth and proliferation of tumor cells [36]. AKT-JNK/p38/ERK has been shown to be involved in the progression of lung cancer and pancreatic cancer [34, 37]. We measured the protein activity of JNK, AKT, ERK and p38. We found that USP44 inhibited the JNK pathway but not the AKT, ERK or p38 pathways. Rescue experiments showed that silencing USP44 expression to promote the proliferation and migration of tumor cells could be blocked by a JNK inhibitor. JNK activation in USP44 knockdown could have been a result of stress-response activation due to chromosome mis-segregation, as reported by Kumar and colleagues [38]. The ubiquitin-proteasome system regulates oncogenic factors post-transcriptionally at the epigenetic level. Studies have shown that important tumor-related factors, such as the epidermal growth factor receptor, sarbox-2, c-myc, and McL-1, are regulated by USPs. However, little is known about the catalytic substrates of USP44. In current study, overexpression of USP44 enhanced the malignancy of glioma by stabilizing tumor-promoter securing [12]. USP44 can induce the genesis of prostate cancer cells partly by stabilizing EZH2 [39]. Therefore, further studies are needed to ascertain whether USPP44 regulates a promoter or tumor suppressor in ccRCC.

## Conclusions

USP44 was underexpressed in ccRCC. USP44 overexpression inhibited the proliferation and migration of 786-O cells and Caki-1 cells significantly. The JNK pathway is involved in the way that USP44 regulates proliferation and migration of 786-O cells and Caki-1 cells.

## Supplementary information

**Additional file 1: Figure S2.** Full-length gel images for Fig. 2b,d,j,l. **Figure S3.** Full-length gel images for Fig. 3e,g. **Figure S4.** Full-length gel images for Fig. 4e,h. **Figure S5.** Full-length gel images for Fig. 5a,b,c. **Figure S6.** Full-length gel images for Fig. 6a.

## Data Availability

All data and materials are available.

## Abbreviations

AKT: Protein kinase B
BrdU: 5-bromo-2′-deoxyuridine
CCK: Cell counting kit
ccRCC: Clear cell renal cell carcinoma
DMEM: Dulbecco’s modified Eagle’s medium
FBS: Fetal bovine serum
GEO: Gene Expression Omnibus
JNK: Jun N-terminal kinase
MAPK: Mitogen activated protein kinas
MMP: Matrix metalloproteinase
mTOR: Mammalian target of rapamycin
RCC: Renal cell carcinoma
RT-PCR: Reverse transcription-polymerase chain reaction
SAC: Spindle assembly checkpoint
sh: Short hairpin
USP: Ubiquitin-specific protease
