# Supplementary material for: Ubiquitin-specific protease-44 inhibits the proliferation and migration of cells via inhibition of JNK pathway in clear cell renal cell carcinoma
Source: BMC Cancer. 2020 Mar 12;20:214. doi: 10.1186/s12885-020-6713-y (PMC7068999; doi:10.1186/s12885-020-6713-y)
Supplement: Supplementary file 1 — Additional file 1: Figure S2. Full-length gel images for Fig. 2b,d,j,l. Figure S3. Full-length gel images for Fig. 3e,g. Figure S4. Full-length gel images for Fig. 4e,h. Figure S5. Full-length gel images for Fig. 5a,b,c. Figure S6. Full-length gel images for Fig. 6a. [file 12885_2020_6713_MOESM1_ESM.zip › Supplementary Figure S5R7.pdf]

# Western blots for supplementary Fig.5

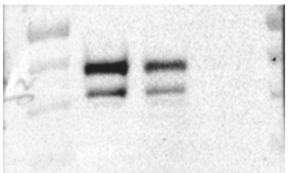

Fig5A p-JNK

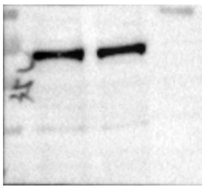

Fig5A p-AKT

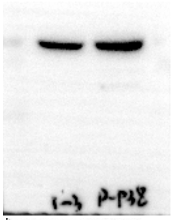

Fig5A p-p38

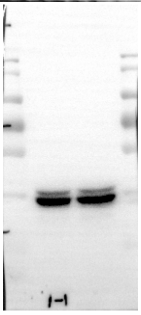

Fig5A p-ERK

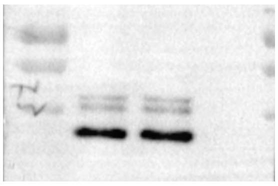

Fig5A β-ACTIN

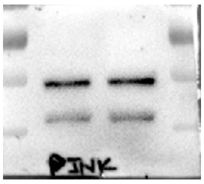


Fig5B JNK

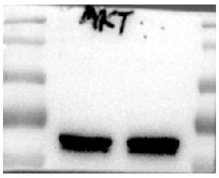


Fig5B AKT

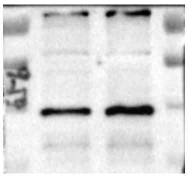

Fig5B p38

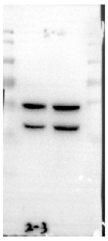

Fig5A JNK

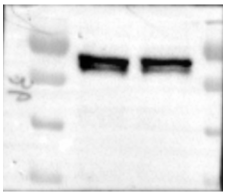

Fig5A AKT

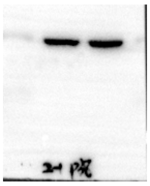

Fig5A p38

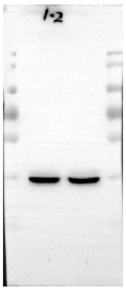

Fig5A ERK

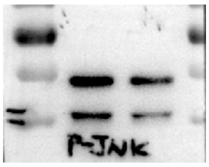


Fig5B p-JNK

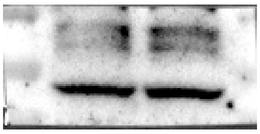

Fig5B p-AKT

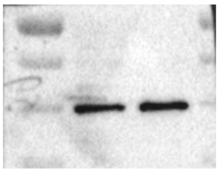

Fig5B p-p38

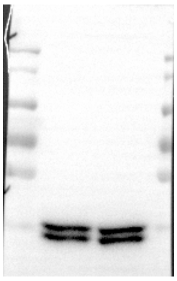

Fig5B p-ERK

## Original uncropped gels of representative Western blot image relateing to indicated Figures

# Western blots for supplementary Fig.5

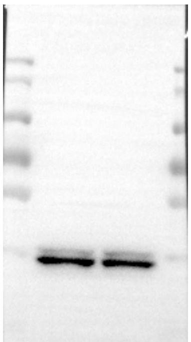

Fig5B ERK

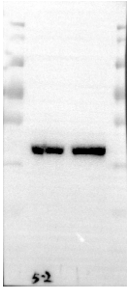

Fig5B β ACTIN

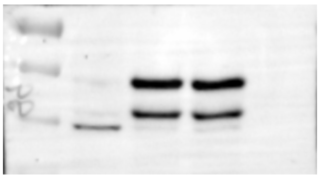

Fig5C p-JNK

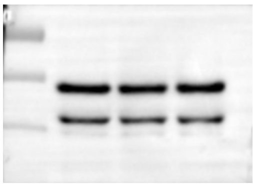

Fig5C JNK

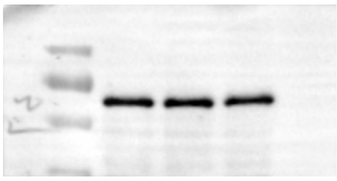

Fig5C p-AKT

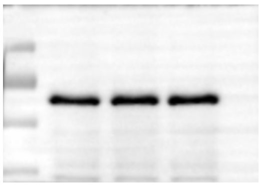

Fig5C AKT

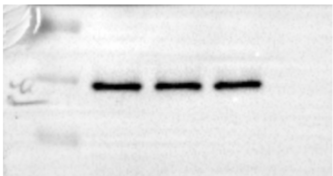

Fig5C p-p38

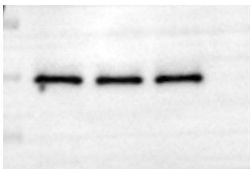

Fig5C p38

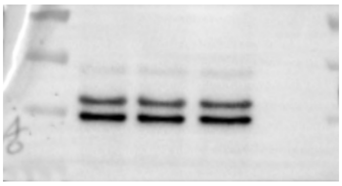

Fig5C p-ERK

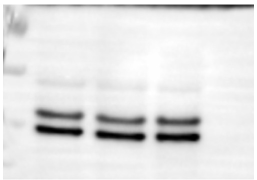

Fig5C ERK

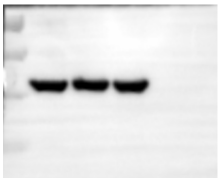

Fig5C β-ACTIN

**Original uncropped gels of representative Western blot image relateing to indicated Figures**
