# Supplementary material for: Ubiquitin-specific protease-44 inhibits the proliferation and migration of cells via inhibition of JNK pathway in clear cell renal cell carcinoma
Source: BMC Cancer. 2020 Mar 12;20:214. doi: 10.1186/s12885-020-6713-y (PMC7068999; doi:10.1186/s12885-020-6713-y)
Supplement: Supplementary file 1 — Additional file 1: Figure S2. Full-length gel images for Fig. 2b,d,j,l. Figure S3. Full-length gel images for Fig. 3e,g. Figure S4. Full-length gel images for Fig. 4e,h. Figure S5. Full-length gel images for Fig. 5a,b,c. Figure S6. Full-length gel images for Fig. 6a. [file 12885_2020_6713_MOESM1_ESM.zip › Supplementary Figure S6R7.pdf]

Western blots for supplementary Fig.6

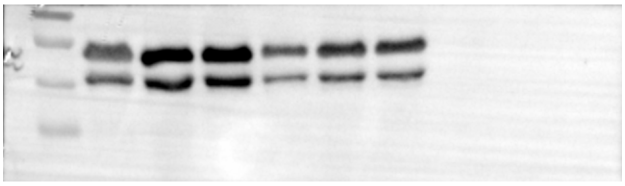

Fig6A p-JNK

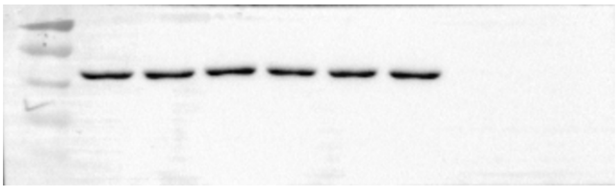

Fig6A β-ACTIN

Original uncropped gels of representative Western blot image relateing to indicated Figures
